# The Liquid Biopsy for Lung Cancer: State of the Art, Limitations and Future Developments

**DOI:** 10.3390/cancers13163923

**Published:** 2021-08-04

**Authors:** Daniel Di Capua, Dara Bracken-Clarke, Karine Ronan, Anne-Marie Baird, Stephen Finn

**Affiliations:** 1 Department of Histopathology, St. James’s Hospital, D08NHY1 Dublin, Ireland; danny.dicapua@hse.ie; 2 Department of Medical Oncology, St. James’ Hospital, D08NHY1 Dublin, Ireland; dara.brackenclarke1986@gmail.com; 3 Faculty of Medicine, University College Dublin, D04V1W8 Dublin, Ireland; karine.ronan@ucdconnect.ie; 4 School of Medicine, Trinity Translational Medicine Institute, Trinity College, D02PN40 Dublin, Ireland; bairda@tcd.ie

**Keywords:** lung cancer, liquid biopsy, circulating tumor DNA, circulating tumor cells, non-small cell lung carcinoma

## Abstract

**Simple Summary:**

During the development and progression of lung tumors, processes such as necrosis and vascular invasion shed tumor cells or cellular components into various fluid compartments. Liquid biopsies consist of obtaining a bodily fluid, typically peripheral blood, in order to isolate and investigate these shed tumor constituents. Circulating tumor cells (CTCs) are one such constituent, which can be isolated from blood and can act as a diagnostic aid and provide valuable prognostic information. Liquid-based biopsies may also have a potential future role in lung cancer screening. Circulating tumor DNA (ctDNA) is found in small quantities in blood and, with the recent development of sensitive molecular and sequencing technologies, can be used to directly detect actionable genetic alterations or monitor for resistance mutations and guide clinical management. While potential benefits of liquid biopsies are promising, they are not without limitations. In this review, we summarize the current state and limitations of CTCs and ctDNA and possible future directions.

**Abstract:**

Lung cancer is a leading cause of cancer-related deaths, contributing to 18.4% of cancer deaths globally. Treatment of non-small cell lung carcinoma has seen rapid progression with targeted therapies tailored to specific genetic drivers. However, identifying genetic alterations can be difficult due to lack of tissue, inaccessible tumors and the risk of complications for the patient with serial tissue sampling. The liquid biopsy provides a minimally invasive method which can obtain circulating biomarkers shed from the tumor and could be a safer alternative to tissue biopsy. While tissue biopsy remains the gold standard, liquid biopsies could be very beneficial where serial sampling is required, such as monitoring disease progression or development of resistance mutations to current targeted therapies. Liquid biopsies also have a potential role in identifying patients at risk of relapse post treatment and as a component of future lung cancer screening protocols. Rapid developments have led to multiple platforms for isolating circulating tumor cells (CTCs) and detecting circulating tumor DNA (ctDNA); however, standardization is lacking, especially in lung carcinoma. Additionally, clonal hematopoiesis of uncertain clinical significance must be taken into consideration in genetic sequencing, as it introduces the potential for false positives. Various biomarkers have been investigated in liquid biopsies; however, in this review, we will concentrate on the current use of ctDNA and CTCs, focusing on the clinical relevance, current and possible future applications and limitations of each.

## 1. Introduction

Lung cancer (LCa) remains a leading cause of death worldwide, contributing to approximately 18.4% of cancer-related deaths globally [1]. Importantly, while LCa rates have been falling in men, rates have been rising in women with LCa now the leading cause of cancer mortality in both sexes in many countries [2].

Non-small cell lung cancer (NSCLC) has seen a rapid rise in the identification of various genetic drivers over the last decade, and more importantly, the development of targeted therapies and immunotherapy. Molecular testing for many targetable genetic markers is now considered routine practice in the management of advanced adenocarcinoma, including activating mutations in epidermal growth factor (EGFR), BRAF, Her2, MET exon 14 splicing, NTRK, ERBB2, KRAS and rearrangements of anaplastic lymphoma kinase (ALK) and ROS proto-oncogene1 (*ROS1*) [3,4,5].

Unfortunately, a significant proportion of people present with advanced disease (stage III/IV) and surgery may not be indicated [6]. In such cases, the only tissue that may be available for molecular testing are small needle core biopsies or cytology specimens, which is not always sufficient due to lack of tumor material. This would necessitate a further re-biopsy, which carries an 8.4% complication risk [7] and overall major complications rate of 5.7% for CT-guided core biopsies and 4.4% for FNA-based biopsies [8]. Furthermore, biopsies in many LCa cases are not feasible outright or at re-biopsy due to tumor location or performance status [9].

Acquisition of sufficient material for diagnosis, subtyping and molecular subtyping is not always straightforward and can lead to delays in “real-world” practice [10]. The need for complete diagnostic workup is counterbalanced by the need to proceed promptly to treatment, especially in cases deemed borderline for therapy. Therefore, any technique enabling a safe and/or convenient, more rapid completion of the diagnostic workup is clearly both beneficial and applicable to routine clinical practice.

**Figure 1:**
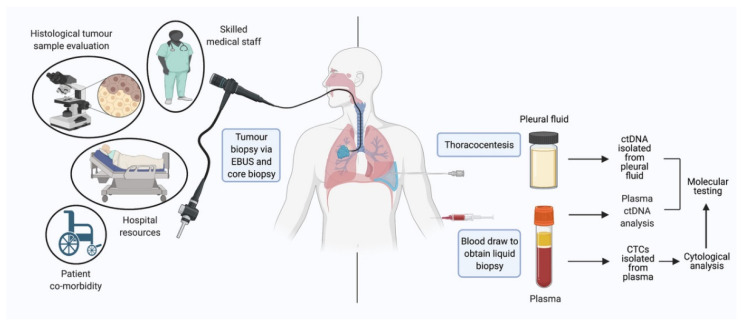
Technical considerations when acquiring tumor tissue and liquid biopsy specimens and preparing samples for diagnostic and monitoring purposes in NSCLC. Core tissue biopsy and EBUS requires careful consideration of patient factors such as comorbidity and frailty, and may be complicated by procedure risk. Technically skilled medical staff and significant healthcare resources are required for such procedures, which limits their availability. Acquisition of sufficient material for diagnosis, subtyping and molecular subtyping is not always straightforward and can lead to delays in “real-world” practice. Sample collection for liquid biopsy is minimally invasive, via blood draw or pleural fluid analysis, technically straightforward and minimizes patient risk. NSCLC = non-small cell lung carcinoma; EBUS = endobronchial ultrasound; CTCs = circulating tumor cells; ctDNA = circulating tumor DNA.

Liquid biopsies could possibly fulfil this role by allowing for a wide range of cytological and molecular assessment modalities through minimally invasive techniques. Samples for liquid biopsies consist of various bodily fluids (most frequently peripheral blood) to obtain intact circulating tumor cells (CTCs) or tumor macromolecular products including, circulating cell-free DNA (cfDNA), circulating tumor DNA (ctDNA), circulating miRNA, exosomes, tumor educated platelets, and various others. These products could subsequently be used in diagnostics, prognostication, monitoring of treatment and assessing mutational status as the disease evolves (Figure 1).

Given the relative convenience of liquid biopsy in addition to its inherent ability to sample tumor heterogeneity, liquid biopsy is highly attractive for assessing tumor biology and molecular status both at a single time point (e.g., at diagnosis or relapse) as well as longitudinally. It is clearly established that Lca, as with most cancers, demonstrates genomic instability with the progressive acquisition of genetic alterations (including point mutations, chromosomal instability and epigenetic alterations) albeit at varying rates– resulting in the development of a genetically diverse disease [11,12,13,14]. Clonal evolution of these pathways and the resulting Darwinian selection which occurs following therapy represents a major mechanism for the development of treatment resistance and disease progression – with the concept of the mixed or paradoxical response being well described [15,16,17,18]. Furthermore, given the increasing role of targeted therapy, the importance of monitoring the molecular profile of each individual patient’s disease to identify resistance mechanisms is likely to increase. Thus, any and all testing modalities which offer a safe and convenient means to assess Lca biology are likely to be of significant clinical utility.

In this review, we aim to discuss the underlying technologies behind, and approaches to liquid biopsy, the current and likely future clinical role of techniques to assess liquid biopsy and probable future directions in lung cancer.

### 1.1. Lung Cancer Screening and Surveillance/Response Assessment

A central element to oncological care is the ongoing assessment of disease status – both follow up after radical therapy with surgery or (chemo-) radiotherapy and of those with metastatic disease receiving active therapy. This is generally based on clinical assessment, serial radiologic imaging and, dependent on the underlying tumor type, and tumor marker monitoring. In the case of Lca, serum biomarkers are not generally used and surveillance is clinical/radiological [19]. The role of routine Lca screening has traditionally been controversial—although the recently published NELSON trial by de Koning et al. [20] demonstrated a significant reduction in Lca specific mortality with CT surveillance of 24%. As regards Lca surveillance (i.e., following therapy with curative intent), serum biomarkers are not generally used and screening is predominantly radiological [19]. While radiologic surveillance (primarily by CT imaging) is effective, it exposes the patient to radiation, has significant resource implications and frequently begets further assessments/investigations on the grounds of the so-called “incidentaloma”—Orme et al. [21] demonstrating a 40% rate of incidental findings on research CTs with 6.2% generating a clinical action. Serological and bronchogenic biomarker panels have successfully been used to further stratify lung cancer risk for patients with radiological findings [22,23,24]. However, when used as a primary screening modality, theses biomarkers fail to identify many patients with lung cancer who do not meet the eligibility for radiological screening [25].

The potential incorporation of liquid biopsy techniques into Lca screening algorithms both for routine population screening and following definitive therapy represents a most attractive approach and an area of active investigation with promising early results [26,27]. Extrapolating from this, should liquid biopsy be determined to have favorable sensitivity and specificity characteristics, it is entirely conceivable that it may become a primary means for screening with radiologic/histologic techniques used for confirmation. The use of liquid biopsy ctDNA for cancer surveillance is an area of active research with positive early data in colorectal and pancreatic cancers [28,29,30]. A further potential role for liquid biopsy techniques is in the clarification of equivocal radiologic lesions – a not unusual situation and frequently encountered as a first manifestation of relapse (for which histologic confirmation would generally be sought, in the absence of fairly unequivocal radiology). In this setting, a suspicious radiologic lesion in the setting of concordant liquid biopsy findings would likely be sufficient evidence for the diagnosis of relapse, potentially saving both time, resources and an invasive procedure with the intrinsic complication risks thereof. Thus, liquid biopsy techniques are likely to play a significant role in both the initial diagnosis of and surveillance for Lca while, potentially at least, reducing the need for imaging and tissue sampling. Aside from its potential use in surveillance post radical therapy, the role of liquid biopsy in those with advanced disease receiving active therapy is at least as great. Assessment of disease response to systemic therapies is a critical part of ongoing oncologic management and, especially in cases of borderline performance status due to disease and/or visceral crisis, one which requires ongoing and sometimes rapid answers – due to the potential to lose the therapeutic “window”. Additionally, while radiologic assessment is the cornerstone of cancer restaging, certain disease sites (particularly bone and leptomeningeal metastases) and therapies (especially immunotherapy with its potential for pseudoprogression) can render conventional radiological assessment equivocal [31,32,33]. In light of this, access to sensitive, specific and targeted biomarker testing would be of great benefit to Lca surveillance and therapy monitoring. While data for Lca surveillance are limited, there is clear evidence for the prognostic value for liquid biopsy in this setting – lending credence, with an excellent underlying scientific rationale, to its utility in response assessment [34,35,36,37,38]. Dawson et al. [39] demonstrated the use of liquid biopsy for the ongoing assessment of people with breast cancer on active therapy with a clear association between rising ctDNA levels (CTCs also having an association, albeit less clear) and radiologically confirmed disease progression with rising ctDNA levels associated with progressive disease in 89% of cases examined. The same process and rationale should apply, in principle, to Lca and is an area of active investigation [40]. Thus, the relative ease of obtaining a liquid biopsy combined with its inherent ability to assess the systemic tumor burden makes it extremely attractive as an adjunct means of restaging Lca—and likely other malignancies also.

### 1.2. Liquid Biopsy in Lca Diagnosis and Molecular Assessment

While conventional histological or cytological specimens are the gold standard for Lca diagnosis, these are not always feasible to achieve safely or conveniently in clinical practice. Furthermore, assessment of suspect fluids (primarily pleural effusions and cerebrospinal fluid—CSF) is frequently important in routine clinical practice and complicated by an appreciable miss-rate by conventional cytology, especially in histologies other than bronchogenic adenocarcinoma and/or necrotic tumors [41,42,43]. Pleural effusion assessment is of particular clinical relevance in the setting of an ipsilateral effusion associated with otherwise early-stage disease (a not uncommon clinical situation) and the need to differentiate a reactive/parapneumonic effusion (frequently encountered in association with a Lca—especially in the setting of post-obstructive collapse/consolidation) from a genuinely malignant effusion. Liquid biopsy is potentially of significant adjunctive use in this setting. Guo et al. [44,45,46] amongst others demonstrated the feasibility of extracting Lca molecular markers, such as ctDNA, from pleural fluid supernatant (achieving greater yields than conventional cellular pellet techniques).

## 2. Circulating Tumor Cells

CTCs are intact tumor cells circulating in peripheral blood, which are shed from either the primary tumor or metastatic deposits with a short half-life of approximately 1 to 2.4 h [47]. CTCs, when present, vary greatly in number from patient to patient, and represent a small fraction of nucleated cells within blood. In NSCLC specifically, CTC counts have been found to range from undetectable, to 1281 CTC/mL [48]. Factors that contribute to the variation in CTC levels in blood have not been determined, and likely include extrinsic factors, such as CTC isolation method. The relationship to clinical stage remains controversial with some studies showing a correlation between more advanced stage NSCLC and increased CTC numbers [49,50], while others have reported no significant relationship between CTC number and stage [51,52,53].

CTCs in blood provide a promising avenue as they could directly be used to diagnose Lca using long established practices used in cytology, while also representative of tumor heterogeneity and a source of genetic material for examination. This is effectively already in practice in non-blood body fluids such as pleural fluid and endobronchial ultrasound FNAs, where cytological material with sufficient cellularity is used for molecular assessment when tissue is not available. While these current cytological methods are beneficial to the patient by providing diagnostic information and possible therapeutic options, involvement of the pleural cavity or peribronchial lymph nodes represents advanced stage disease. Detection of CTCs in peripheral blood could allow for detection of earlier stage cancer [54]. A summary of the studies assessing CTCs in lung cancer is given in Table 1.

### 2.1. Capture/Isolation

One of the most significant challenges regarding clinical utilization of CTCs is reliable capturing technologies and separation from background leukocytes. Currently, CellSearch (Veridex LLC) is the only capture assay FDA approved for use in monitoring of CTCs in colon, breast and prostatic cancer. This method uses a ferrofluid containing magnetic nanoparticles coated with anti-epithelial adhesion molecule (EpCAM), and quantification is achieved using fluorescent probes [63]. This method has a significant limitation in that EpCAM non-expressing cells are not detected, such as those undergoing epithelial-mesenchymal transition (EMT). A recent study assessing EMT markers in NSCLC CTCs found that more cells expressed a purely mesenchymal or mixed epithelial-mesenchymal transcriptome (55% and 77.8% of cases, respectively) compared to purely epithelial (27.8% of cases). While the study population size was small, this highlights a possible limitation with EpCAM-based capture technologies in NSCLC [52].

Isolation by size of epithelial tumor cells (ISET) is another technology available, whereby size, and not cell surface protein expression, is the main discriminator of CTCs. This method uses variably sized filters to separate cells based on size and deformability. Similarly, Screencell^®^ is another filter-based system which discriminates based on cell size, rather than surface markers [64,65]. While it is capable of retrieving EpCAM negative tumor cells, small CTCs would be lost thereby affecting quantification and further limiting a potential source of material for further analysis [66]. A novel method, currently under review by the FDA, is the Parsortix system. This system is based not only on the size of CTCs, but also the increased rigidity of CTCs. There has been limited experience with NSCLC; however, one recent study reported increased CTC yields over the CellSearch system in matched samples [56].

Various other technologies have been developed to extract CTCs from blood. One promising system uses microvortices and inertial forces, exploiting differences in fluid dynamic characteristics between CTCs and native blood cells to separate them [67,68]. One group has also enriched CTCs within samples prior to CTC extraction by using leukapheresis to obtain a sample enriched in nucleated cells in blood samples. This method resulted in a 30 fold increase in CTCs extracted in breast and prostate cancer compared to the CellSearch system [69].

### 2.2. Clinical Use

In both non-treated and post-treatment in the advanced NSCLC setting, CTCs appear to be of prognostic relevance. Two meta-analyses have shown that the simple presence of CTCs in people with advanced NSCLC was a poor prognostic indicator of progression free survival [PFS] and overall survival (OS) [58,70]. However, due to the wide variation in CTC isolation methods, definitive CTC cut-off values for survival and risk stratification are unclear. In the post-chemotherapy setting, detection of fewer than 5 CTCs per 7.5 mL of peripheral blood were associated with longer PFS and found to be a strong predictor of OS [57]. This relationship has also been observed in people harboring an activating mutation in EGFR and treated with targeted therapy, where fewer than 5 CTCs per 7.5 mL of blood post-treatment was associated with a significant increase in time to treatment failure and PFS [71].

At present, there have only been a handful of studies assessing CTCs specifically in early stage NSCLC. One study using the ISET isolation method, found that 50 CTCs or more (per 10 mL of peripheral blood) identified preoperatively by cytomorphological methods, corresponded to a significantly worse overall and disease free survival in people with resectable stage I or II NSCLC [26]. A more recent study assessing stage 1 tumors only, found that all patients with increasing CTCs in the postoperative period subsequently relapsed [52]. Furthermore, intra-operative increased levels of CTCs in the central circulation (namely the pulmonary vein) has also been correlated with worse prognosis in terms of OS, disease free survival (DFS), and time to metastasis [55,72,73], although whether this is due to surgical manipulation or specific tumor characteristics is unclear [74]. While it appears that CTCs may help identify early NSCLC patients that will perform poorly post treatment, the exact relationship between CTC levels and patient outcome requires further investigation.

The presence of CTC clusters, as opposed to single cells, appears to also have prognostic benefit. Generally, CTC clusters are defined as more than one CTC within close proximity/adherent, with some authors stratifying clusters by size, with large clusters equating to greater than 5 CTCs [75]. Clusters have also been found to be more prevalent in those with high CTC numbers (greater than 18 CTC per 7.5 mL of blood) [55]. It has been postulated that these clusters may in fact serve as a nidus from which metastatic deposits arise. In the preoperative setting, identification of CTC clusters in peripheral blood was associated with worse PFS [60]. Similarly, a study assessing post-operative patients found that CTC clusters identified in the pulmonary vein were associated with a significantly worse relapse-free survival, whereas survival did not significantly differ in patients with either no CTCs or exclusively single cell CTCs [76]. While the data may be limited, this is an early indication that CTC clusters may be a good clinical marker for identifying patients at risk of relapse. However, many CTC studies did not differentiate between single CTCs and clusters, and larger studies would be required to establish this relationship with greater confidence.

Lung cancer screening is an area in which CTCs have shown some promise. An early study into the usefulness of CTCs as an Lca used the ISET system to isolate CTCs from blood in high-risk patients with COPD, with CTCs confirmed through cytomorphological and immunocytochemical criteria. Of the 168 high risk patients included, 5 were found to have CTCs, and a concurrent low dose CT showed no evidence of lung nodules. On follow up, these 5 patients developed early stage tumors, including 4 adenocarcinomas and 1 squamous cell carcinoma [77]. The AIR study project, led by the same group, attempted to validate the results in a prospective, multicenter study in France. The study failed to confirm results, and in addition, failed to detect CTCs in 13 of 15 people with Lca identified on imaging; however, specificity was high [78]. A possible factor for this was that, while all the ISET system was used for all specimens, handling was not standardized for all centers, and variations in collection, transport and time to processing could have affected the outcome. While this does not exclude CTCs from a future role in Lca screening, it highlights the need for standardized pre-analytical protocols.

Once isolated and quantified, CTCs can subsequently be used in molecular assessment of tumors. EGFR mutations, including the resistance mutation T790M, have been identified through CTC-derived genetic material previously [79]. A recent study attempted to characterize activating mutations in single CTCs using digital droplet PCR (ddPCR). Only 16.7% of known positive EGFR activating mutations in matched tissue samples were detected when one CTC was used, and detection increased marginally to 33.3% when 10 CTCs were used [80]. Another possible application of CTCs is in the detection of genetic rearrangements, with ALK and ROS1 rearrangements successfully identified through fluorescent in situ hybridization (FISH) [81,82]. NGS sequencing of single CTCs isolated from intraoperative central and peripheral blood have demonstrated that many “CTCs” are likely to represent benign epithelial cells. However, a second population of cells was noted on both morphology and sequencing that were aneuploidy and harbored a similar mutational profile to the tissue obtained from the tumor, indicating that CTCs may be a possible surrogate for tumor tissue [61]. These studies serve as a proof of concept that molecular studies can be performed on CTCs; however, there is currently limited data and studies usually include small sample sizes, requiring further exploration.

## 3. Circulating Tumor DNA

cfDNA in peripheral blood originates from normal tissue remodeling with variable contributions by tumor necrosis, apoptosis and potentially through extracellular vesicles [83,84]. cfDNA exists in a nucleosome protected 150–200 base pair sized fragments and has a half-life of approximately 2 h, allowing analysis of the genomic material to reflect the current, real time status of the target(s) in question [85]. The concentration of cfDNA in plasma is typically low (5–10 ng/mL), and the fraction that corresponds to ctDNA can be highly varied and range from as low as 0.1% to 30% of the total cfDNA [86,87].

### 3.1. Methodology Considerations

With such small concentrations of DNA, special consideration is required in sample collection of liquid biopsies for cfDNA or ctDNA. Lysis of nucleated cells within samples, especially lymphoid cells, could release vast amounts of non-tumor DNA, effectively “drowning out” ctDNA and leading to false negatives. For this reason, plasma is preferred over serum, as the clotting process leads to leukocyte lysis [88]. Appropriate collection and storage of samples is also crucial in order to minimize leukocyte lysis. Standard EDTA blood collection tubes are suitable for sample collection; however, samples must be processed ideally within 4 h from collection at room temperate or 24 h at 4 °C, in order to avoid significant blood cell lysis. Alternatively, proprietary collection tubes containing leukocyte stabilizing agents are available, including Streck BCT tubes (Streck Inc., Omaha, NE, USA), PAXgene tubes (Qiagen PreAnalytiX GmbH, Hilden, Germany), and cfDNA collection tubes (Roche Diagnostics GmbH, Mannheim, Germany). These collection tubes are capable of maintaining adequate sample integrity for at least 48 h, and possibly up to a week at room temperature [89,90].

### 3.2. Molecular Testing

The ability to detect ctDNA in a background of “normal” cfDNA poses a significant challenge. Assays need to be sensitive enough to detect the proverbial needle in a haystack, where allelic copies of mutated genes can be very low amongst the total DNA pool [86,87]. Furthermore, cfDNA and ctDNA exist in highly fragmented forms, and assay detection capabilities are required that are robust enough to detect these fragments. Many types of assays have been developed which have shown success in overcoming these challenges and are typically classified in one of two groups; targeted gene detection methods and broad panel/whole genome methods [91]. The targeted detection methods typically have higher sensitivity and are either PCR or NGS-based methods.

PCR-based methods which have shown to have the sensitivity required for analysis of ctDNA include ddPCR and BEAMing [92]. The basis of ddPCR is emulsification of the DNA within a sample into droplets containing approximately one DNA fragment each. Two chromophores are then used to distinguish between target mutation and wild-type DNA, which are detected as the samples are cycled. BEAMing is similar to ddPCR; however, biotinylation is used to bind amplified DNA to magnetic beads, allowing for direct extraction of target DNA [93]. While sensitive and cost effective, PCR-based methods do have significant limitations. Firstly, in the setting of NSCLC, they are not recommended for interrogating ALK and ROS rearrangements [92]. Secondly, PCR-based methods are able to interrogate discreet and known genetic alterations, and are limited in terms of the number of genetic targets each assay can detect. While multiplexing expands the number of targets tested per sample, the ever-increasing number of targetable genetic alterations means that broader methods will be required.

Targeted NGS platforms have been developed to allow for an expanded repertoire of targets tested. Amplicon-based and hybrid-capture-based platforms are available which provide sequencing of specific genetic targets in order to identify any actionable alterations. Amplicon-based NGS consists of using primers to amply specific portions of ctDNA, which are then sequenced. The main advantages of this method is that it requires considerably less starting material [94], and is less expensive than the hybrid-capture method [95]. Hybrid capture NGS involves using DNA or RNA fragments targeting areas of interest to purify ctDNA fragments from the remaining cfDNA. Capture-based methods provide a wider coverage with more consistent data, at the cost of requiring larger initial DNA sample, more laborious workflow, longer turnaround times and greater cost [94]. While advances in amplicon-based NGS have increased reliability and sensitivity, it is limited to known hotspots and panels are less expensive than capture NGS [96,97]. Furthermore, while it is possible to detect gene rearrangements with amplicon-based NGS, this requires the use of circulating tumor RNA (ctRNA), or incorporates multiplex PCR [98]. Hybrid capture NGS is technically more challenging; however, a well-developed and validated system could provide wider coverage, detect a wider array of genetic alterations, and provide more reliable data than amplicon-based NGS [94]. While both methods have distinct merits and limitations, other factors that have to be taken into consideration when adopting these NGS technologies into practice such as specific scope of use, resources and downstream bioinformatic capabilities. An in depth review of NGS on cfDNA samples, and clinical uses has been recently published by Esposito Abate et al. [99].

In a comparison of amplicon NGS with ddPCR, NGS had a high sensitivity for single nucleotide variants, indels and selected rearrangements and showed to have positive percentage agreement of 95%, and a positive predictive value of 100% [100]. When compared to tissue biopsy samples, concordance with NGS findings in liquid biopsy while initially variable, is improving in multiple recent studies [101,102,103,104]. One possibility for discrepancy between tissue and ctDNA is genetic alterations which are missed in the tissue biopsy due to tumor heterogeneity. This is evident in one study where EGFR T790M mutation was detected in ctDNA and not in concurrent tissue biopsy, yet patients subsequently benefited from osimertinib therapy [105]. Furthermore, low levels of ctDNA can lead to false negative results compared to tissue biopsies [104].

NGS platforms are advantageous over digital PCR methods in that they can discern between multiple different genetic alterations within the same targeted genetic locus. Cancer Personalized Profiling by deep Sequencing (CAPP-Seq) is another NGS-based system which uses probes designed from libraries of known NSCLC genetic alterations. These probes are applied to ctDNA and multiple loci are simultaneous amplified, which are then sequenced [106]. This process was further refined by the development of an integrated digital error suppression technology which eliminates stereotypical background errors. When assessed in patient serum samples, ctDNA detection level of this method was 0.004% (4 in 10^5^ cfDNA molecules), with a 90% sensitivity and 96% specificity [107].

### 3.3. Clinical Use

The use of ctDNA to guide clinical management of NSCLC presents many advantages. One of the most significant being that it is a less invasive method with a lower risk of complication for disease monitoring than serial tissue biopsy [8]. While tissue biopsies (and cytological material to an extent) remain the ‘gold standard’ for diagnostics, it remains limited with regard to serial monitoring for the development of resistance mutations or minimal residual disease (MRD) due to associated risks. Furthermore, liquid biopsy-derived ctDNA can be more representative of a heterogeneous tumor or metastatic deposit, and detect actionable targets that may otherwise be missed on a single site tissue or FNA biopsy [11,108]. A summary of studies assessing the utility of ctDNA in lung cancer is provided in Table 2.

The use of ctDNA from peripheral blood in the detection of EGFR mutations in NSCLC is already widely in use [92]. ctDNA analysis has been particularly beneficial in cases where tumor biopsy is not feasible, or where obtaining a tissue biopsy would delay the initiation of treatment [92]. Many studies have shown wide concordance in EGFR mutation status between ctDNA and tissue biopsies [117,121], with some studies showing that concurrent tissue and ctDNA analysis improved detection of molecular alterations, ultimately leading to the identification of increased numbers who may benefit from targeted therapies [113,122]. A recent systematic review was conducted by Wang et al., looking at the diagnostic accuracy ctDNA in detecting EGFR mutation. There were 40 studies included (5995 patients), and pooled sensitivity was found to be 68% (95%CI = 60–75%), and the specificity was 98% (95%CI = 95–99%) [123]. The authors concluded that, while ctDNA is specific, its sensitivity indicates that negative results should be followed up by tissue biopsy if possible.

Aside from initial detection of an *EGFR* mutation, ctDNA analysis techniques have an established role in the detection of resistance mutations to first and second generation *EGFR* TKIs such as T790M [14,124]. Liquid biopsy ctDNA is effective in detecting evidence of the T790M mutation (along resistance mutations in ROS1 and ALK) with comparable accuracy to conventional histologic/cytological samples [111,125,126,127,128]. While perhaps best defined for the *EGFR* T790M mutation, ctDNA techniques appear equally valid in other driver mutations, including *ALK, ROS1* and *NTRK,* with liquid biopsy enabling serial assessment and detection of resistance mutations for these driver mutations [129,130].

Lca screening is another area for which ctDNA is, at least theoretically, extremely attractive based on relative convenience and need for multiple samples over time; however, its role in screening is presently uncertain. While detection of ctDNA in late stage tumors is fairly reliable, the sensitivity of ctDNA detection in early stage tumors remains low (47–50%) [106,131,132] and therefore not adequate for screening purposes. Sensitivity could be improved with larger panels and technology advancements; however, this incurs an increased cost and may not be cost effective for screening. There is also the possibility that ctDNA could be used in conjunction with LDCT in at risk populations. The currently ongoing SUMMIT trial will be assessing ctDNA as a screening method for various malignancies using the GRAIL blood test as well concurrent LDCT in patients at high risk for Lca (NCT03934866).

Currently, there are 2 assay kits approved by the EMA and FDA for the testing of EGFR in liquid biopsies, TheraScreen EGFR RGQ PCR Kit (Qiagen, Hilden, Germany) and Cobas EGFR Mutation test v2 (Roche Diagnostics, Basel, Switzerland). Multiple other platforms have been developed, both PCR-based as well as NGS-based. Unfortunately, while the use of ctDNA is widely used in the detection of EGFR mutations, there no standardization of this process with institutions using varied approaches to conduct testing. To illustrate the variation, one recent survey of seven hospitals in North Eastern Italy found that 2 hospitals used the Cobas EGFR Mutation Test v2, five hospitals used Easy EGFR (Diatech Pharmacogenetics, Jesi, AN, Italy), 2 centers employed confirmation tests based on NGS and ddPCR, and 2 centers also performed EGFR testing on pleural fluid [133]. The significance of using different mutation detection methodologies is unclear as both Cobas and TheraScreen kits have been shown to have high concordance rate of EGFR mutation detection with tissue/cytological specimens [134]; however, direct comparison between various detection methods are lacking.

### 3.4. ctDNA in Other Fluids—Pleural and CSF

Liquid biopsies traditionally have focused on peripheral blood; however, ctDNA can be detected in malignant plural effusions (MPE) in patients with advanced Lca. Cytological examination of cells and tissue fragments from MPE is already routine practice for diagnosis [135]. Additionally, molecular analysis using formalin fixed paraffin embedding (FFPE) tissue blocks or cell pellets generated from centrifuged cytology samples show high concordance with tissue biopsy and are routinely being used as source genetic material for molecular analysis [136,137]. However, this cellular component is often insufficient for molecular workup due to minimal cellularity or small tumor cell fraction. A potential solution is the use of supernatant fluid from cytological specimen preparation, which is typically discarded, as a source of ctDNA. When compared to FFPE blocks or pellets, supernatant fluid has shown a very high concordance in mutation detection [138], and a superior concordance with mutational status assessed in tissue biopsy [136]. MPE liquid biopsies may also provide a better representation of tumor heterogeneity than tissue biopsy. Multiple studies that found discordant positive findings in MPE compared to tumor tissue biopsies of primary lung tumors, showed a substantial portion of those were actually novel tumor mutations not represented in the original tumor biopsy [136,137,139,140,141].

Central nervous system (CNS) metastases (including both parenchymal brain metastases and leptomeningeal carcinomatosis) represent one of the most feared and clinically devastating complications of malignancy with Lca being particularly associated with CNS involvement [142,143,144,145,146,147]. The blood–brain barrier presents challenges for detection of ctDNA from CNS metastases as it inhibits the excretion of ctDNA into peripheral blood [131]. The use of cerebrospinal fluid (CSF)-derived ctDNA has already been a focus for the detection of CNS primary tumors, as these tumors shed little, if any ctDNA into the peripheral circulation [148,149]. Additionally, liquid biopsies of CSF may provide genetic material needed for the diagnosis and management of patients with CNS and leptomeningeal metastasis (LM). In one study, 35 patients with known meningeal carcinomatosis (MC) from various solid organ tumors, the majority of which were Lca, were assessed using CSF cytology, neuroimaging and CSF liquid biopsy for the assessment of cancer associated mutations. CSF cytology identified malignant cells in 25 patients, neuroimaging identified MC in 22 patients; however, all CSF biopsies identified cancer associated mutations [150]. Furthermore, data suggested that ctDNA derived from CSF is superior to that derived from plasma for the identification of tumor mutation profiles, likely owing to the fact that ctDNA constitutes a much larger fraction of cfDNA in CSF with higher detection rates in the CSF of patients with known LM compared to synchronously assayed plasma. Notably, CSF analysis demonstrated improved rates of actionable mutation (e.g., *EGFR*, *ALK*, *ROS1*, *BRAF*) and resistance mutation (e.g., *EGFR* T790M mutation) detection —driver genes being detected in 100%, 84.6% and 73.1% of samples comprising CSF cell-free DNA (cfDNA), CSF precipitates, and plasma, respectively in one study [111,151,152]. The detection of such driver mutations along with associated resistance mutations is of particular clinical import given both the considerably better prognosis of such patients (due to the possibility of targeted therapy) and the relatively high rates of CNS involvement along with the varying CNS penetration and activity of various TKIs [124,153,154,155,156]. In addition, a study by Ying et al. [157], where ctDNA obtained from CSF in patients with LM provided identification of more unique genetic alterations and higher maximum allelic fraction compared to plasma ctDNA. Thus, liquid biopsy techniques represent a clinically useful, convenient and safe methodology to both assess malignant involvement of fluid spaces and perform requisite molecular profiling for actionable mutations/resistance mechanisms. They may also have a role in assessment of treatment response (e.g., ctDNA no longer being detectable following therapy) and in early assessment of relapse/recurrence prior to disease being appreciable cytologically.

### 3.5. ctDNA Limitations

With the development of large panel NGS platforms, liquid biopsies can be assessed for a wide range of tumor driving and targetable genetic alterations. However, cfDNA in peripheral blood only contains a small fraction of ctDNA with the bulk of the remaining DNA originating from hematopoietic cells [86,87]. As with all cells, hematopoietic progenitor cells acquire somatic mutations, and thus establish distinct non-neoplastic clonal populations of circulating cells, a phenomenon referred to clonal hematopoiesis of uncertain clinical potential (CHIP) [158,159]. The exact incidence of CHIP is unclear, but does rise with increasing age with an estimated 10–20% of people over the age of 70 showing evidence of CHIP [160]. Mutations in clonal populations have been identified in a wide variety of genes, with some mutations characteristic of CHIP (*DNMT3A*, *TET2*, *ASXL1*, and *JAK2*), and others in known targetable genes commonly seen in solid tumors [160,161]. TP53 mutations are commonly identified in CHIP with variants previously reported in solid tumors [162,163,164]. More specifically related to Lca, EGFR mutations have been noted in a study on healthy controls; however, these represented different variants than those observed in NSCLC and are not thought to be oncogenic [165]. CHIP clearly raises an issue in terms of clinical interpretation of liquid biopsies, as false positive results can potentially be detected in large proportion of patients and in a wide array of genes.

Various methodologies are being employed to filter out CHIP-related mutations from genuine ctDNA-derived mutations. The most straightforward method is conducting parallel studies on nucleated white blood cells to identify CHIP mutations and exclude them from those discovered in plasma panels [166]. While this method is technically feasible and simple to conduct, it effectively doubles costs and reduces the cost effectiveness of ctDNA analysis Chabon et al. [162] recently developed a multi factorial machine-learning-based method specifically targeting NSCLC tumors. They noted that non-ctDNA fragments were of larger size and less fragmented that ctDNA fragments and discriminated based on size criteria. The authors then used machine learning to identify frequently recurring genetic alterations occurring in NSCLC and CHIP in patients with high risk of developing NSCLC and definite invasive carcinoma. They successfully identified tumor-derived mutations, leading to the diagnosis of early stage NSCLC without the need for concurrent individualized white blood cell panels.

The rapid development of technologies and possible applications of ctDNA in the literature is certainly encouraging, but has also highlighted a potential issue. As previously mentioned, there is a wide array of platforms currently in use, from sample collection to molecular analysis. Molecular methodologies make use of numerous different platforms, which are primarily PCR- or NGS-based. Therefore, comparing studies is not always straight forward and a consensus or standardization of some methods may be required.

The use of ctDNA for molecular profiling also suffers from false negatives in a cohort of patients deemed to be “nonshedders” and consists of patients with known tumor driver mutations identified on tissue biopsy, but not detected in ctDNA. The shedding of ctDNA is thought to be due to tumor biology where larger tumors, presence of necrosis and tumor vascularity have been associated with increased levels of ctDNA [167,168]. In regard to primary early stage lung adenocarcinoma features which have been associated with increased ctDNA shedding are increased tumor burden, solid morphology, necrosis and increased mitotic activity [110]. Tumors lacking these features may not be shedding ctDNA into circulating blood, or minimally shedding ctDNA at levels below current detection limits. Interestingly, in one study, patients with EGFR T790M mutation who were pre or post-treatment nonshedders have been found to have increased OS and post-progression survival despite osimertinib failure [169].

### 3.6. Minimal Residual Disease

ctDNA is currently being investigated as potential marker for minimal residual disease (MRD) in patients who have undergone therapy for NSCLC. There is a demonstrable decrease in ctDNA in patients undergoing tumor resection for NSCLC [170,171], and monitoring for an increase could indicate an evolving relapse. In one prospective study, postsurgical monitoring of ctDNA following curative NSCLC primary resection showed that over half of patients had detectable ctDNA post treatment, while 72% of patients had detectable ctDNA prior to relapse. Additionally, the detection of ctDNA, predated radiological evidence of relapse by a median of 5.3 months [109]. Improving the detection of early LCa and disease monitoring is one of the objectives of the TRACERx trial (NCT01888601) through longitudinal assessment of changes in tumor genetics and identifying novel biomarkers and genetic profiles of NSCLC [172]. While requiring further development, a liquid biopsy approach for MRD would be very beneficial to patients as it would provide a minimally invasive modality which could be used to identify patients at higher risk of early relapse. 

Some of the current limitations of MRD monitoring using ctDNA reflect assay design. More sensitive assays typically rely on whole-genome or exome sequencing with development of personalized tumor-informed patient specific panels. This provides improved sensitivity; however, it is more costly, and also limits the ability of detecting de novo resistance/oncogenic alterations [173]. Another method involves sequencing 128 genes commonly mutated in LCa (including known driver genes), thus avoiding the need for an individualized tumor informed panels. This method is more cost effective and shows a high specificity in one small study assessing its use in MRD for LCa [109]. As in any other application of ctDNA, CHIP presents a significant pitfall that could lead to false positives, and methods used to account for this are described elsewhere in this review.

## 4. Future Clinical Directions

Following increasing availability of NGS techniques and greater understanding of the fundamental molecular pathways in cancer, there has been an increasing focus on tumor subtyping and assessment for actionable mutations. This has led, at least in part, to cancers being classified based on their molecular profiles rather than on tissue of origin and histologic subtype. A further result of this has been the establishment of so-called molecular tumor boards (MTBs) in which fundamental molecular pathways responsible for malignant transformation are identified in an individual patient and determining treatments likely to directly target these pathways. MTBs have been proliferating significantly in recent years and are likely to continue to expand and enter more routine practice [174]. Liquid biopsy is likely to greatly facilitate this as it enables both convenient initial tumor molecular assessment and longitudinal molecular assessment. Thus, resistance mechanisms may be elucidated and, theoretically at least, directly targeted.

**Figure 2:**
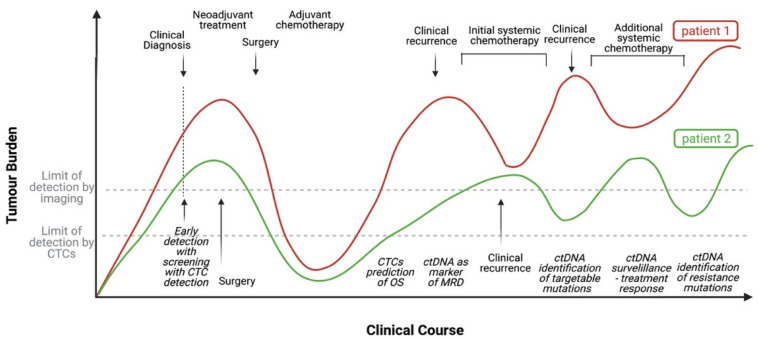
Graph demonstrating the potential roles of ctDNA and CTCs in the clinical course of a patient with NSCLC compared with standard approaches to patient care. Patient 1 demonstrates standard approaches to screening, diagnosis and monitoring of a patient with NSCLC. Patient 2 demonstrates the potential utility of ctDNA and CTCs in the disease course of a patient with NSCLC.

Although limited by cost, technical challenges and availability, liquid biopsy techniques have definite and clinically relevant applications to the management of LCa—both early—and late stage. Thus, while it is highly likely and predictable that liquid biopsy will play a major future role in the diagnosis, response assessment and ongoing surveillance of LCa, supportive data are, at present, relatively limited. Perhaps the greatest challenge facing healthcare services is the rising cost and complexity of interventions (including the requirement for detailed molecular diagnosis) with the frequent need to repeat this assessment over time during the treatment course—to say nothing of the resultant complication risk and the burden on people with the disease. In light of this, liquid biopsy techniques would appear to offer an ideal combination of convenience and safety while still providing detailed molecular information and at least reducing, if not fully obviating, the need for invasive and technically complex tissue sampling. Thus, the authors feel that liquid biopsy represents probably the major future direction in LCa diagnosis, assessment, surveillance and, probably, screening—and will likely play a similar role in other malignancies (Figure 2). That said, further trials with greater numbers as well as “real-world” data will be required to verify this along with cost and time reductions which are the likely result of ongoing technical improvement and economies of scale.

## 5. Conclusions

Liquid biopsies, in particular, ctDNA, have already made it to clinical use, albeit with a limited role. The technical capability to assess the relatively small amounts of ctDNA or CTCs in blood and other fluids has been quickly improving and becoming more reliable. One drawback to this rapid advancement is that there is a lack of standardization, and comparison between studies is often difficult. In regard to ctDNA, the number of targets and types of genetic alterations that we can reliably assess is ever increasing due to NGS. However, detecting low ctDNA levels such as in early LCa, post treatment or detecting MRD is still a major challenge. With increasing sensitivity and an expanding repertoire of assessable targets, clonal hematopoiesis has emerged as a factor which needs to be accounted for in the application of ctDNA. The precise pathophysiological role and implications of CTCs will require further clarification. However, in the right clinical context, CTCs appear to provide helpful information regarding risk stratification for patients with LCa. The liquid biopsy, in its many different forms, has the potential to be greatly beneficial to patients, not only through better diagnosis, prognostication and monitoring, but also by decreasing the amount of invasive procedures required.

## Figures and Tables

**Table 1:** Summary of published studies assessing the role of CTCs in lung cancer.

Author	Number of Patients	Isolation Method	Main Findings
Crosbie et al. [55]	33	CellSearch	CTC clusters associated with increased overall CTC counts. High CTC counts (>18/7.5 mL blood) associated with reduced DFS and OS.
Hofman et al. [26]	208	ISET	>50 circulating non-hematological cells were associated with shorter OS and DFS.
Janning et al. [56]	127	Parsotrix and CellSearch	Parsotix detected at least 1 CTC in 61% of samples compared to 32% for CellSearch. CTCs were PD-L1^+^ in 47% of cases, PD-L1^+^ and PD-L1^−^ in 47% and PD-L1^−^ in 7%. Increase in PD-L1^+^ CTCs seen in disease progression.
Jin et al. [52]	45	CanPatrol	CTC counts increased with disease progression. CTCs with mesenchymal features more abundant in tumors >2 cm. higher post-operative CTCs associated with tumor progression.
Krebs et al. [57]	101	CellSearch	Stage IV NSCLC had higher CTCs than stage III NSCLC. <5 CTCs pre-chemotherapy were associated with longer PFS and better OS.
Li et al. [53]	174	Negative enrichment	CTCs detected in 79.3% of patients. CTCs showed a higher diagnostic efficacy compared to serum tumor markers.
Lindsay et al. [58]	550	CellSearch	CTC counts of >2/7.5 mL blood was associated with reduced PFS, and >5/7.5 mL of blood with worse OS.
Manjunath et al. [59]	60	Microfiltration	CTC clusters noted in 41.2% of NSCLC patients. No CTC clusters identified in patients with radiographically benign lesions.
Murlidhar et al. [60]	36	OncoBean chip	CTC clusters associated with worse prognosis. Clusters displayed genotypic characteristics of therapeutic resistance.
Tamminga et al. [61]	31	CellSearch	CTCs were detected more frequently and in greater numbers from pulmonary vein compared to radial artery. Post-operative decrease in CTCs noted in blood from radial artery, but not the pulmonary vein. 1.2% of cells isolated showed aneuploidy, indicating majority of cells likely epithelial cells.
Wei et al. [50]	73	Nano-enrichment, direct visualization	Average CTC numbers were 5.7/7.5 mL of blood and decreased to 2.4/7.5 mL of blood after chemotherapy. <5 CTC/7.5 mL of blood showed better PFS. EGFR mutations were associated with greater number of CTCs.
Xu et al. [62]	20	Microfludic chip	75% of patients had detectable CTCs. Successful WES on single isolated CTC, with detection of 6 new mutations, concordant with surgical specimen.

Abbreviations: DFS: disease free survival; OS; overall survival; PFS; progression free survival; WES: whole exome sequencing.

**Table 2:** Summary of published studies assessing the role of ctDNA in lung cancer.

Author	Number of Patients	Platform	Main Findings
Chaudhuri et al. [109]	94	CAPP-seq (NGS)	Detectable ctDNA post-treatment preceded radiological evidence of progression in 72% of cases. Of the patients that relapsed, 94% had detectable ctDNA after treatment with curative intent.
Cho et al. [110]	36	PANAmutyper (PCR)	Factors associated with higher ctDNA in plasma included higher pathological tumor stage, nodal metastasis, solid adenocarcinoma subtype, tumor necrosis, greater tumor volume and frequent mitoses.
Li et al. [111]	26	WGS	Driver genes detected in all CSF ctDNA samples. 92.3% of patients had higher allele fractions in CSF than CSF precipitates or plasma. EGFR T790M was detected in CSF of 30.4% samples from patients who progressed on TKI.
Oxnard et al. [105]	216	BEAMing	Plasma detection of T790M was 70% sensitive. OOR and PFS were similar T790M positive tumors detected through plasma ctDNA or biopsy.
Papadopoulou et al. [112]	171	NGS	49% of NSCLC patients had at least 1 mutation detected at diagnosis by NGS. 86.1% concordance in clinically relevant mutations between ctDNA and tissue biopsy.
Sabari et al. [113]	210	ResBio ctDx-Lung	ctDNA detection lower in patients on systemic treatment. High concordance of ctDNA detected oncogenic drivers with tissue detection (91.6%).
Tailor et al. [114]	33	SureSelect All Exon V5 + UTR	Patients with malignant nodules showed a significantly higher number of somatic mutations. 82% of malignant lesions identified through mutational analysis.
Tsui et al. [115]	50	Tam-Seq PCR, digital PCR	Low levels of EGFR mutations in TKI naïve patients resulted in better PFS and OS. Pre-treatment mutations in both EGFR and TP53 correlated with worse prognosis. Progression without T790M mutation resulted in worse survival.
Uchida et al. [116]	288	NGS	EGFR exon 19 deletion sensitivity was 50.9% and specificity was 98.0%. L858R mutation sensitivity was 51.9% and specificity was 94.1%.
Weber et al. [117]	199	Cobas EGFR test	91% concordance of EGFR mutations between tissue and plasma ctDNA samples. Six EGFR mutations detected in ctDNA samples only.
Yang et al. [118]	103	Gardant360	Poor survival if >3 mutations detected in ctDNA
Zhang et al. [119]	27	NGS	Overall ctDNA and tissue concordance for driver gene mutations was 85.2%, sensitivity and specificity was 87.0% and 75%, respectively. Concordance reached 100% in cases of boney metastasis and/or concurrent TP53 mutations.
Zhao et al. [120]	111	Mutant-enriched PCR	EGFR mutation concordance between paired plasma and tissue samples was 71.2%. Sensitivity was higher for poorly differentiated tumors (77.8%) compared to well differentiated (20%) and moderately differentiated (19%) tumors.

Abbreviations: CSF: cerebrospinal fluid; EGFR: epidermal growth factor; OOR: objective response rate; PFS; progression free survival; NGS; next generation sequencing; TKI: tyrosine kinase inhibitors; OS; overall survival.
